# The caffeine-binding adenosine A_2A_ receptor induces age-like HPA-axis dysfunction by targeting glucocorticoid receptor function

**DOI:** 10.1038/srep31493

**Published:** 2016-08-11

**Authors:** Vânia L. Batalha, Diana G. Ferreira, Joana E. Coelho, Jorge S. Valadas, Rui Gomes, Mariana Temido-Ferreira, Tatiana Shmidt, Younis Baqi, Luc Buée, Christa E. Müller, Malika Hamdane, Tiago F. Outeiro, Michael Bader, Sebastiaan H. Meijsing, Ghazaleh Sadri-Vakili, David Blum, Luísa V. Lopes

**Affiliations:** 1 Instituto de Medicina Molecular, Faculdade de Medicina de Lisboa, Universidade de Lisboa, Portugal; 2 Max Planck Institute for Molecular Genetics, Berlin,Germany; 3 Department of NeuroDegeneration and Restorative Research, University Medical Center Goettingen, Waldweg 33, 37073 Göttingen, Germany; 4 Instituto de Farmacologia e Terapêutica, Faculdade de Medicina do Porto, Universidade do Porto, Portugal; 5 Faculdade de Ciências de Lisboa, Universidade de Lisboa, Portugal; 6 Max-Delbrück-Center for Molecular Medicine (MDC), Berlin, Germany; 7 PharmaCenter Bonn, Pharmazeutische Chemie I, Pharmazeutisches Institut, University of Bonn, Bonn, Germany; 8 Department of Chemistry, Faculty of Science, Sultan Qaboos University, Muscat, Oman; 9 Univ. Lille, Inserm, CHU Lille, UMR-S 1172, Alzheimer & Tauopathies, Lille, France; 10 Max Planck Institute for Experimental Medicine, Goettingen, Germany; 11 CEDOC, Centro de Estudos de Doenças Crónicas, Lisbon, Portugal; 12 Charité-University Medicine Berlin, Germany; 13 Institute of Biology, University of Lübeck, Germany; 14 MassGeneral Institute for Neurodegenerative Disease, Massachusetts General Hospital, Boston, MA, United States of America

## Abstract

Caffeine is associated with procognitive effects in humans by counteracting overactivation of the adenosine A_2A_ receptor (A_2A_R), which is upregulated in the human forebrain of aged and Alzheimer’s disease (AD) patients. We have previously shown that an anti-A_2A_R therapy reverts age-like memory deficits, by reestablishment of the hypothalamic-pituitary-adrenal (HPA) axis feedback and corticosterone circadian levels. These observations suggest that A_2A_R over-activation and glucocorticoid dysfunction are key events in age-related hippocampal deficits; but their direct connection has never been explored. We now show that inducing A_2A_R overexpression in an aging-like profile is sufficient to trigger HPA-axis dysfunction, namely loss of plasmatic corticosterone circadian oscillation, and promotes reduction of GR hippocampal levels. The synaptic plasticity and memory deficits triggered by GR in the hippocampus are amplified by A_2A_R over-activation and were rescued by anti-A_2A_R therapy; finally, we demonstrate that A_2A_R act on GR nuclear translocation and GR-dependent transcriptional regulation. We provide the first demonstration that A_2A_R is a major regulator of GR function and that this functional interconnection may be a trigger to age-related memory deficits. This supports the idea that the procognitive effects of A_2A_R antagonists, namely caffeine, on Alzheimer’s and age-related cognitive impairments may rely on its ability to modulate GR actions.

Excessive glucocorticoid production associated with chronic or severe stress impairs hippocampal neuronal function and predisposes the organism to neurodegeneration^1^. Release of cortisol from the adrenal cortex is under tight regulation of this hypothalamic–pituitary–adrenal (HPA) axis. The hippocampus plays a crucial role in regulating HPA axis^2^ and excessive glucocorticoid production disrupts the regulatory circuit that connects the hippocampus and the hypothalamus.

Age-related disorders are associated with downregulation of glucocorticoid receptors (GR) in the hippocampus, and subsequent desensitization of the regulatory feedback to the hypothalamus^3^. Accordingly, in a large study of elder humans aged 50–70 years, elevated salivary levels of cortisol were found to be correlated with poor cognitive function^4^. Increased glucocorticoid activity has also been associated with greater hippocampal atrophy and memory impairment in the elderly^3^. This is probably a consequence of dendritic retraction and hippocampal dysfunction that we have shown to occur upon chronic stress^1^. Moreover, higher cortisol levels have been also associated with more rapid Alzheimer’s disease (AD) progression^5^ and systemic administration of glucocorticoids or stress were shown to potentiate memory impairments, hippocampal damage, β-amyloid formation and Tau accumulation in transgenic AD mice^6^,^7^,^8^.

In the recent years, multiple lines of evidence have suggested an association between adenosine modulation and stress response. In particular, activation of the adenosine A_2A_ receptor (A_2A_R) was shown to contribute to the stress response by inducing corticosterone secretion^9^ and by mimicking GR effects^10^. Moreover, we have recently shown that oral administration of an A_2A_R antagonist restores morphological, behavioral, and synaptic deficits induced by HPA-axis dysfunction in rodents^1^. As observed for HPA axis, we and others have demonstrated that A_2A_ receptors are dysregulated in the rat or human brain upon aging and AD^11^,^12^,^13^.

There is a striking parallel between A_2A_R over-activation/over-expression and impaired GR receptor function, as evidenced by the similar ability of A_2A_R and GR antagonists to improve cognitive deficits as well as to mitigate amyloid and Tau pathologies reminiscent of AD^14^,^15^,^16^,^17^. Altogether, such observations strongly suggest that A_2A_R over-activation and GR dysfuntion are key events in age-related hippocampal deficits and raise the possibility that both pathways might be interconnected.

In the present study, we provide the first demonstration of the instrumental impact of A_2A_R modulation of GR function, a mechanism never hypothesized before. We specifically report that A_2A_R overexpression in forebrain neurons is sufficient to promote HPA-axis dysfunction, namely loss of plasmatic corticosterone circadian oscillation, and reduced GR hippocampal levels, both being age-related phenotypes^18^. Further, we show that A_2A_R activation modulates GR-induced deficits in hippocampal synaptic plasticity, increasing susceptibility to GR activation. Finally, we demonstrate that A_2A_R modulation impacts GR nuclear translocation and transcriptional activity.

## Materials and Methods

### Animals

All experimental procedures were carried strictly within the rules of the Portuguese official veterinary department, which complies with European Directive 2010/63/EC and the Portuguese law transposing this Directive (DL 113/2013); and approved by the *Instituto de Medicina Molecular* Internal Committee and the Portuguese Animal Ethics Committee (*Direcção Geral de Veterinária*). Environmental conditions were kept constant: food and water ad lib, 21 ± 0.5 °C, 60 ± 10% relative humidity, 12 h light/dark cycles, 2 to 3 animals per cage. The animals were killed by decapitation after anesthesia under halothane atmosphere. Male WT Sprague-Dawley and Tg(CaMKII-hA_2A_R) rats were used in the 8–14 week-old age range for all the experiments described.

### Generation and maintenance of transgenic animals

**Figure 1:**
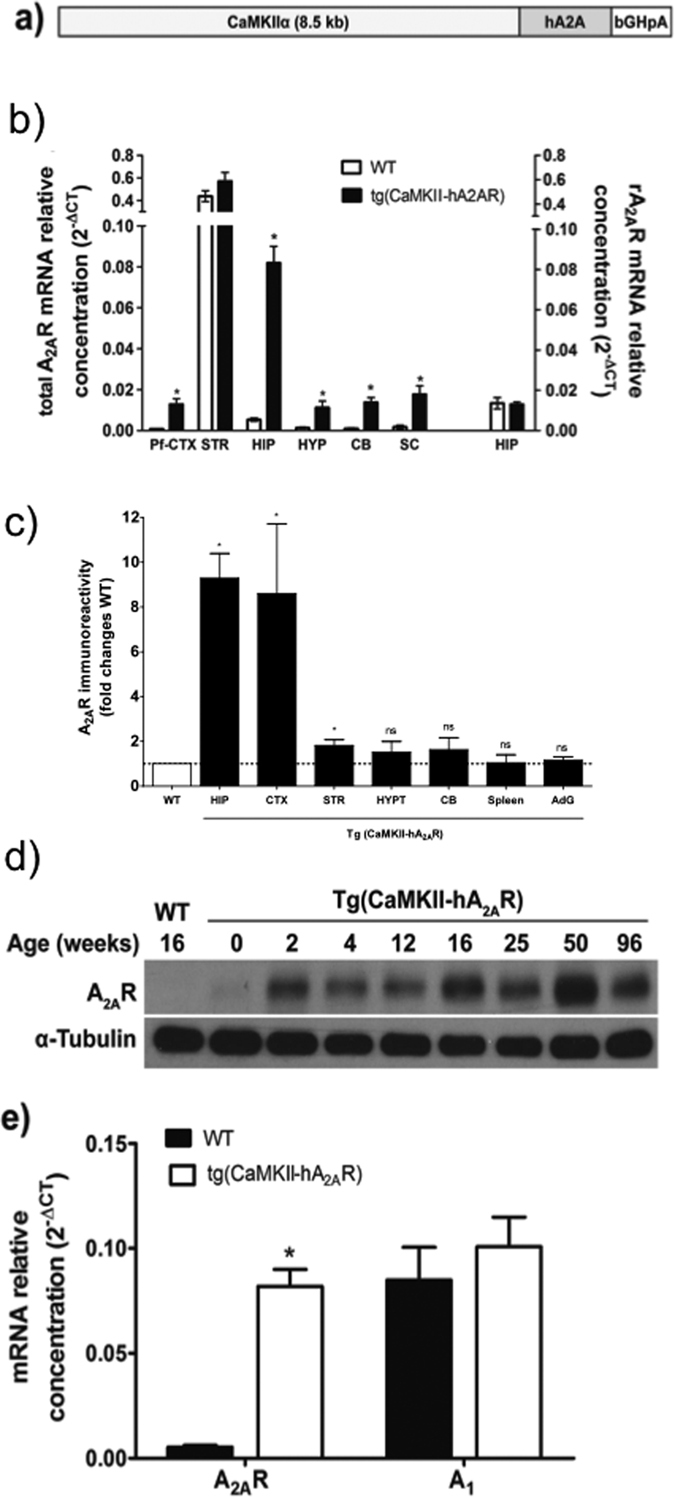
Tg(CaMKII-hA_2A_R) rats overexpress hA_2A_R in forebrain areas. (**a)** Construct used to generate Tg(CaMKII-hA_2A_R) rats. (**b,c)** Animals present an overexpression of total A_2A_R in the forebrain confirmed by qPCR and Western blotting. The endogenous (right axis) rA_2A_R mRNA levels were not modified in the hippocampus as assessed with specific rat A_2A_primers. (**d)** A_2A_R protein levels increase from 2 weeks old onwards in the hippocampus and **(e)** no changes were detected in adenosine A_1_R mRNA levels for 12–14 weeks old animals. Results were analysed using unpaired Student’s t-test for each gene/brain area, *P < 0.05 compared to WT.

Transgenic rats overexpressing the human adenosine A_2A_ receptor (A_2A_R) under the control of the CaMKIIα promotor, tg(CaMKII-hA_2A_R), were generated by microinjection of a linearized DNA construct into the male pronucleus of Sprague–Dawley rat zygotes with established methods^19^. The construct contained a full-length human A_2A_R cDNA cloned into an expression vector with the 8.5 kb mouse CaMKIIα promoter^20^ and a polyadenylation cassette of bovine growth hormone (see Fig. 1a). Sprague Dawley wild type (WT) male rats were used as controls. Genotyping: Transgenic rats were identified by PCR (30 cycles, 58 °C annealing temperature) of their genomic DNA isolated from ear biopsies by the use of the transgene-specific primers CaMKII-hA2A and rat β-actin primers as an internal control (Invitrogen, see Table 1). Breeding efficiency and litter size was not affected in tg(CaMKII-hA_2A_R) animals. The average weight of the animals was also similar between WT 282.9 ± 37.7 g and tg(CaMKII-hA_2A_R) 286.7 ± 22.8 g at the age tested.

### RNA extraction and quantitative real-time PCR analysis (RT-qPCR)

Total RNA was extracted and purified using the RNeasy Lipid Tissue Mini Kit (Qiagen) for tissue samples and with NucleoSpin RNA kit (Macherey-Nagel) for neuronal cultures. RNA quality was assessed by NanoDrop 2000 (Thermo Scientific) analysis (A260/A280 ≈ 2; 260/235 >1.8). Total RNA (2 μg) was reverse-transcribed using random primers and SuperScript™ First-Strand Synthesis System for RT-PCR (Invitrogen). RT-qPCR analysis was performed on a ABI 7900 HT (Applied Biosystems) using a home mix consisting of 100 mM Tris pH 8.3, 6 mM MgCl_2_, 1 mg/ml BSA, 4 mM dNTPs, 0.66x SYBR Green and 1x ROX reference dye. PPIA (cyclophilin A) and β-actin were used as reference genes for human tissues whereas PPIA, Rpl13A (ribosomal protein L13A) and Pgk1 (phosphoglycerate kinase 1) were used for rat tissues and PPIA (cyclophilin A) for primary cultures. The relative expression of target genes was determined by the comparative CT method^21^.

### Corticosterone quantification

Blood was collected from the tail in animals previously handled to minimize stress and without anesthesia at two different time points, 8 AM, and 8 PM as in^1^. The plasma was isolated by centrifugation at 2000 *g*, 4 °C for 15 min and corticosterone quantified by radioimmunoassay using the rat corticosterone ^3^H kit (MP Biomedicals), according to the manufacturer’s protocol.

### Dissection and tissue collection

After decapitation the brain was rapidly removed and the hippocampi were dissected free in ice-cold Krebs solution composed of (mM): NaCl 124; KCl 3; NaH_2_PO_4_ 1.25; NaHCO_3_ 26; MgSO_4_ 1; CaCl_2_ 2; and glucose 10, previously gassed with 95% O_2_ and 5% CO_2_, pH 7.4). One hippocampus was used for electrophysiological recordings, the remaining brain was separated by areas (Hippocampus, cortex, cerebellum, striatum, hypoyhalamaus) and rapidly frozen in liquid nitrogen for further analysis.

### Behavioural assessments

10–14 weeks old WT and tg(CaMKII-hA_2A_R) rats treated either with vehicle or KW 6002, were first handled for 5 days before testing in the behavior assays. The Y-maze was performed in a two-trial recognition test in a Y-shaped maze with 3 arms (each with 35 cm length x 10 cm width x 20 cm height), angled at 120°; on the first trial (learning trial), the animal explored the maze for 10 min with only two arms opened (start and other arm); after 1 h, the animal is re-exposed to the maze for 5 min (test trial) with the novel arm available, the preference for the novel arm is considered a measure of short-term reference memory. The number of transitions was used to evaluate motor performance. The maze was cleaned with a 70% ethanol solution between each animal. Rat tracings during the learning task were continuously monitored by an automated tracking system (Smart 2.5, PanLab, Barcelona) and the time spent exploring each arm was quantified.

### Electrophysiological recordings

Slices (400 μm thick) were obtained from the same animals used for CORT analysis and behavior testing, with a McIlwain tissue chopper, left to recover for at least 1 h in Krebs solution and field excitatory postsynaptic potentials (fEPSPs) were recorded as previously described^1^ in the CA1 *stratum radiatum*. Long term potentiation (LTP, 100 Hz, 1s) was recorded as previously described at 32 °C with a constant flux of 3 mL/min^1^. Whenever indicated, drugs were preincubated at 32 °C. The intensity of the stimulus was maintained during the induction protocol. LTP was quantified as the % of change in the average slope of the fEPSP measured during the 5 data points immediately preceeding the induction of LTP, comparing to the fEPSP measured from 46 to 60 min (5 data points of 8 averages each) after LTP induction. In each individual experiment, the same LTP-inducing paradigm was delivered to each pathway. Hippocampal slices were incubated with dexamethasone 100 nM (at 32 °C), for the time periods indicated (20′ and 60′), always followed by 60 min resting prior to recording, time necessary to ensure nuclear translocation (as controled in cell cultures) and gene-dependent effects. Antagonists (50 nM SCH58261; 100 nM RU486) were applied 15–20 min before treatment and agonists (CGS21680, 30 nM) at the same time.

### Sample preparation

Nuclear/cytoplasmic fraction enrichment was performed by differential centrifugation. Samples were homogenized with a 29G syringe and centrifuged at 1000 *g* for 10 min. The supernatant is the cytoplasmic fraction; the pellet was resuspended in 100 μL of sucrose buffer (0.32 M sucrose, 50 mM Tris, pH 7.6), homogenized and centrifuged again to ensure a minimum contamination with cytoplasm. 150 μL of 1.5x sample buffer (350 mM Tris, 30% glycerol, 10% SDS, 600 mM dithiothreitol and 0.012% bromophenol blue, pH 6.8) were added to the nuclear fraction and 15 μL were used for immunoblot detection. The cytoplasmic fraction was prepared with 20 μL of sample and 5 μL of 5x sample buffer. Tissue homogenates of WT and tg(CaMKII-hA_2A_R) were prepared from frozen samples. Briefly samples were homogenized by sonication in immunoprecipitation-assay (RIPA) buffer (50 mM Tris, 1 mM EDTA, 150 mM NaCl 0.1% SDS, 1% NP 40, pH 8.0)^22^. Protein was quantified using the BioRad Protein DC assay based on Lowry^23^. The appropriate volume of sample was completed with sample buffer.

### Western Blotting

Samples were denatured by heating to 95 °C for 5 min or at 70 °C for 30 min for A_2A_R detection. Samples and molecular weight markers were resolved by SDS-PAGE (8% or 10% for resolving and a 5% for stacking gels) in denaturing conditions and electro-transferred to PVDF membranes (Millipore). Membranes were blocked with 5% non-fat dry milk in TBS-T (Tris buffer saline with 0.1% Tween-20, 200 nM Tris, 1.5 M NaCl). After washing with TBS-T, membranes were incubated with primary antibody in TBS-T with 3% BSA. Secondary antibody incubation was in 5% non-fat dry milk in TBS-T. Primary antibodies were rabbit GR specific M20 (1:750/1:1000 sc-1004, Santa Cruz Biotechnology), rabbit lamin A/C specific (1:2000, #2032, Cell Signaling), rabbit pan-cadherin specific (1:20000, abcam ab6529) rabbit αTubulin specific (1:2000, abcam, ab4074), mouse GAPDH specific (1:1000, ambion, AM4300) and mouse A_2A_R specific (1:2000, Upstate/Millipore - 05-717), secondary antibodies conjugated with horseradish peroxidase were goat, rabbit, or mouse specific antibodies (Santa Cruz Biotechnology, Heidelberg, Germany). Chemiluminescence detection was performed with ECL-PLUS western blotting detection reagent (GE Healthcare) using X-Ray films (Fujifilm). Optical density was determined with Image-J software.

### Cell culture

N1E-115 mouse neuroblastoma cells (CRL-2263) were cultured in Dulbecco’s modified Eagle’s medium (DMEM) without pyruvate supplemented with 10% (v/v) fetal bovine serum (FBS), 100 U/ml penicillin-streptomycin, and 2 mM L-glutamine (Gibco). Cells were plated into 6-well plates for 24 h to reach 60% confluence before transfection with Exgene 500 (Euromedex). Briefly 4 μg of pGL3(GRE)3_TK_Luc (GRE_Luc) plasmid (kindly given by Dr. Philippe Lefevbre, Inserm U1011) were mixed in 400 μL of non-supplemented DMEM with 20 μL of Exgene 500 (the mix volume/well) and incubated for 15 min at RT. Cells were incubated for 3 h with the transfection mix before completing the volume to 3 mL. Drug treatments were performed 24 h after transfection in two technical replicates. Primary neuronal cultures. Cortical neurons from 18 days Sprague Dawley rat embryos (Harlan, Barcelona) were cultured according to Valadas^24^. Briefly, the embryos were collected in Hanks’ balanced salt solution (HBSS) and rapidly decapitated. Meninges and white mater were removed and whole cortices were fragmented and cells were isolated by trypsinization in HBSS Ca^2+^/Mg^2+^ (1 mM/1 mM, 0.025% trypsin) and centrifuged at 200 rpm. Cells were washed with HBSS Ca^2+^/Mg^2+^ supplemented with 10% FBS and resuspended in Neurobasal medium. Cells were plated on poly-L-lysine-coated coverslips in 6-well plates at density of 1 × 10^6^ cells/well. Neurons were grown in Neurobasal medium with 2% B-27 supplemented with 25 μM glutamate, 0.5 mM glutamine and 2 U/mL penicillin/streptomycin, in the absence of any positive selection for neurons. The medium was replaced at day 4 (without glutamate). Drug treatments were performed at day 8, 1 h after replacing the medium by Neurobasal without B27. All cells were kept in a 5% CO_2_ humidified incubator at 37 °C.

### Drug treatments

Cell treatments were performed as in Valadas^24^. Drug treatments were vehicle matched to drugs, with a max DMSO of 0.001%. Briefly, mouse neuroblastoma N1E115 cells were treated with dexamethasone 100 nM for 24 h; antagonists (SCH58261 10–100 nM, KW6002 30 nM and RU486 100 nM) were applied 15–20 min before treatment and agonists (CGS21680, 10–50 nM) were co-applied with dexamethasone. After treatment cells were washed in ice-cold PBS and processed for luciferase assay. Primary neuronal cultures were treated with dexamethasone 100 nM for different periods of time 0, 5, 10, 15, 30, 60, 90 min; the A_2A_R antagonist SCH58261 (50 nM) was applied 15–20 min before dexamethasone. After treatment, cells were washed in ice-cold PBS and resuspended in 200 μL of sucrose solution (0.32 M sucrose, 50 mM Tris, pH 7.6) supplemented with protease inhibitors (Roche). Hippocampal slices incubated with dexamethasone 100 nM (at 32 °C), for the time periods indicated (20′ and 60′), always followed by 60 min resting prior to recording, time necessary for nuclear translocation (as controled in cell cultures). Antagonists (50 nM SCH58261; 100 nM RU486) were applied 15–20 min before treatment and agonists (CGS21680, 30 nM) at the same time. *In vivo* therapy: KW6002 (istradefylline, a selective A_2A_R antagonist) or vehicle were orally administered in the drinking water (5 mg/kg/day) to WT male rats as described^1^.

### Luciferase assay

Luciferase activity was evaluated with the luciferase assay system (Promega) according to the manufacture’s procedure. Briefly, N1E115 cells were lysed in 150 μL luciferase cell culture lysis reagent for 15 min at 4 °C. The supernatant was collected after 2 min, centrifuged at 12,000 *g* at 4 °C and 5 μL were used for the assay, each technical replicate was assayed in duplicate. Luciferase activity was measured on a Mithras Microplate Reader LB 940 (Berthold Technologies).

### Drugs

The A_2A_R selective antagonist 2-(2-furanyl)-7-(2-phenylethyl)-7H-pyrazolo[4,3-*e*][1,2,4]triazolo[1,5-*c*]pyrimidin-5-amine (SCH58261), the A_2A_R selective agonist 4-[2-[[6-amino-9-(*N*-ethyl-β-D-ribofuranuronamidosyl)-9*H*-purin-2-yl]amino]ethyl]benzene propanoic acid (CGS21680) and the GR antagonist (11β,17β)-11-[4-(dimethylamino)phenyl]-17-hydroxy-17-(1-propynyl)-estra-4,9-dien-3-one (RU486) were purchased from Tocris Cookson, UK. These drugs were diluted in the assay solution from 5 mM or 10 mM (for RU486) stock aliquots made in DMSO stored at −20 °C. The GR agonist (11β,16α)-9-fluoro-11,17,21-trihydroxy-16-methylpregna-1,4-diene-3,20-dione,9α-fluoro-16α-methyl-11β,17α,21-trihydroxy-1,4-pregnadiene-3,20-dione,9α-fluoro-16α-methylprednisolone (dexamethasone) was purchased from Sigma (Spain), diluted from 10 mM stock in DMSO and stored at −20 °C. The A_2A_R selective antagonist, (*E*)-8-[2-(3,4-dimethoxyphenyl)vinyl]-1,3-diethyl-7-methyl-3,7-dihydropurine-2,6-dione (KW6002, istradefylline) was synthesized according to a published procedure^25^. The purity of the product was determined by HPLC analysis coupled to electrospray ionization mass spectrometry and was greater than 98%. For *in vitro* assays a fresh 10 mM stock solution in DMSO was prepared, used only for 1 week and stored at −20 °C. All other reagents used were of the highest purity available either from Merck, Germany or Sigma Aldrich, Spain.

### Statistics

Values presented are mean ± SEM of n experiments. To test the significance of the differences between groups in Western blotting experiments, unpaired Student’s t-test was used. To compare within slice drug treatments in LTP, paired Student’s t-test was used. In all other experiments, when comparing 3 or more groups a one-way ANOVA was used, followed by a Bonferroni’s Multiple Comparison *post hoc* test. For the analysis of the primary neuronal cultures and corticosterone levels, a two-way ANOVA repeated measures test was used. Values of P < 0.05 were considered to be statistically significant.

## Results

### Overexpression of A_2A_R in forebrain neurons impairs HPA-axis and plasticity, rescued by an *in vivo* anti-A_2A_R therapy

We have previously demonstrated that HPA-axis function and GR hippocampal levels could be restored through blockade of A_2A_R activation^1^. We now tested if the forebrain A_2A_R overexpression – similar to what is found in aged and AD human brain - could be sufficient to drive HPA-axis dysfunction and hippocampal synaptic impairments. We generated transgenic rats that selectively overexpress human A_2A_R in neurons under the control of the CaMKIIα promoter [Tg(CaMKII-hA_2A_R); Fig. 1a] that display cognitive impairments^26^. As expected, expression of A_2A_R was achieved in forebrain areas (Fig. 1b), mainly in the hippocampus and cortex, though we also detected a slight increase A_2A_R mRNA levels in other areas of the nervous system (Fig. 1b). Peripheral expression assessed in the spleen and adrenal glands did not reveal significant changes in A_2A_R protein levels (Fig. 1c). We have observed a temporal profile of protein overexpression in the hippocampus from 2 week-old onwards reaching a plateau at 12 week-old with a 4.9 fold increase in immunoreactivity (Fig. 1d), which is comparable to levels found in physiological aging^27^. Importantly, there was no change in adenosine A_1_ receptor levels in the hippocampus of Tg(CaMKII-hA_2A_R) animals (Fig. 1e).

**Figure 2:**
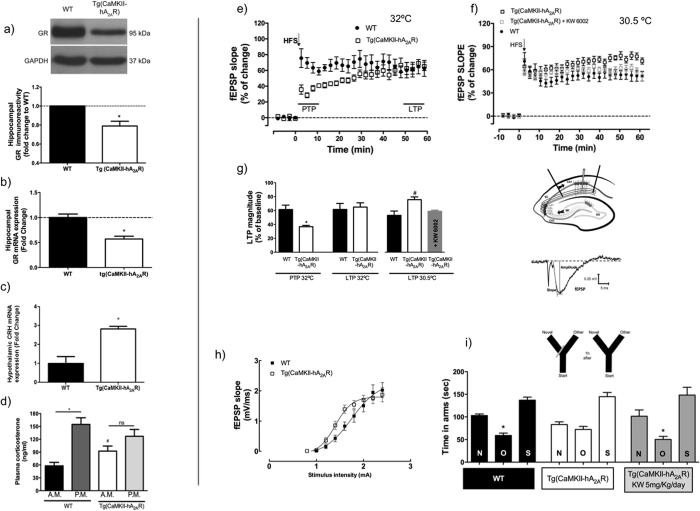
Neuronal overexpression of adenosine A_2A_ receptor (A_2A_R) disrupts HPA-axis function. (**a)** GR protein and **(b)** mRNA levels are decreased in the hippocampus (n = 5) of Tg(CaMKII-hA_2A_R) compared to WT animals, and (**c)** CRH mRNA levels are increased in the hypothalamus (n = 5) of Tg(CaMKII-hA_2A_R) compared to WT animals, calculated using a unpaired Student’s t-test. (**d)** Corticosterone levels evaluated at 8 AM and 8 PM are elevated in Tg(CaMKII-hA_2A_R) and do not oscillate in a circadian manner (n = 6–9); Results were analysed with a two-way ANOVA followed by a Bonferroni *post hoc*. *P < 0.05 compared to WT, ^#^P < 0.05 compared with WT at AM. High frequency stimulation (HFS: 100 Hz, 1s) was used to evaluate synaptic plasticity in hippocampal rat slices according to scheme depicted in lower panel (f). (**e)** At 32 °C, Tg(CaMKII-hA_2A_R) animals present a lower post-tetanic potentiation (PTP) and no effect on long term potentiation (LTP). (**f)** At 30.5 °C, Tg(CaMKII-hA_2A_R) animals present higher LTP magnitude which was rescued by one-month oral administration of an A_2A_R selective antagonist, KW 6002 (5mg/Kg/day). LTP and PTP magnitudes were quantified in (**g**). *P < 0.05 unpaired Student’s t test, compared to WT; and are presented as mean ± SEM of (n = 6–11) experiments; ^#^P < 0.05 using one-way ANOVA, followed by a Bonferroni *post hoc* and (**h**) Input/Output (I/O) curve corresponding to fEPSP slope evoked by different stimulation intensities (0.6 – 3 mA). There is a shift to the left in the curve from Tg(CaMKII-hA_2A_R) animals. (**i**) Short-term reference memory, using the modified Y-maze test. Impairments in Tg(CaMKII-hA_2A_R) animals were rescued by oral KW6002 administration (n = 10–16). Results were analysed with a one-way ANOVA followed by a Bonferroni *post hoc* and are presented as mean ± SEM of n experiments. *^,#^P < 0.05 compared to Novel (N) arm.

We found that A_2A_R overexpression triggered a decrease in GR protein and mRNA levels in the hippocampus (Fig. 2a,b), as well as an increase in CRH mRNA in the hypothalamus, compared to WT animals (Fig. 2c). Additionally, transgenic animals present higher basal levels of corticosterone in the morning (AM) and a loss of the corticosterone circadian oscillation characteristic of WT rats (Fig. 2d). These features are typical of aged animals^18^. In order to understand the impact of A_2A_R overexpression in hippocampal function, fEPSP recordings were performed in the CA1 pyramidal neurons, while stimulating the Schaffer collaterals projections.

Hippocampal slices from [tg(CaMKII-hA_2A_R)] have a decreased post-tetanic potentiation (PTP), whereas long-term potentiation (LTP) was not significantly changed (Fig. 2e,g) compared to WT animals (at 32 °C). To unmask any effect hidden by endogenous adenosine release - which inhibits LTP- we have decreased the adenosine endogenous levels^28^, by lowering the perfusion temperature to 30.5 °C. In these conditions, we were able to reveal an overexcitability of LTP (Fig. 2f,g) in [tg(CaMKII-hA_2A_R)] animals, similar to what we previously described in the aged hippocampus^29^. Accordingly, the input-output curve from Tg(CaMKII-hA_2A_R) rats is shifted to the left compared to WT animals (Fig. 2h). We then investigated if *in vivo* long-term therapy with an A_2A_R blocker would revert synaptic impairments induced by A_2A_R overexpression. The treatment of [tg(CaMKII-hA_2A_R)] rats for 1 month with the selective A_2A_R antagonist, KW6002 [5 mg/kg/day, orally, in the same dose we have shown to rescue GR expression upon stress^1^], rescued LTP amplitude in [tg(CaMKII-hA_2A_R)] to values close to those of WT animals (Fig. 2e,g). Accordingly, when tested for short-term reference memory, using the modified Y-maze test, Tg(CaMKII-hA_2A_R) animals performed worse than wild type (WT), revealing no preference for the novel arm. This effect was rescued by one-month oral tratment with KW6002 (5 mg/kg/day) (Fig. 2i). We found no changes on the total number of transitions, which could compromise the tests and indicate striatum-associated secondary effects.

### Overexpression of A_2A_R increases hippocampal susceptibility to dexamethasone

**Figure 3:**
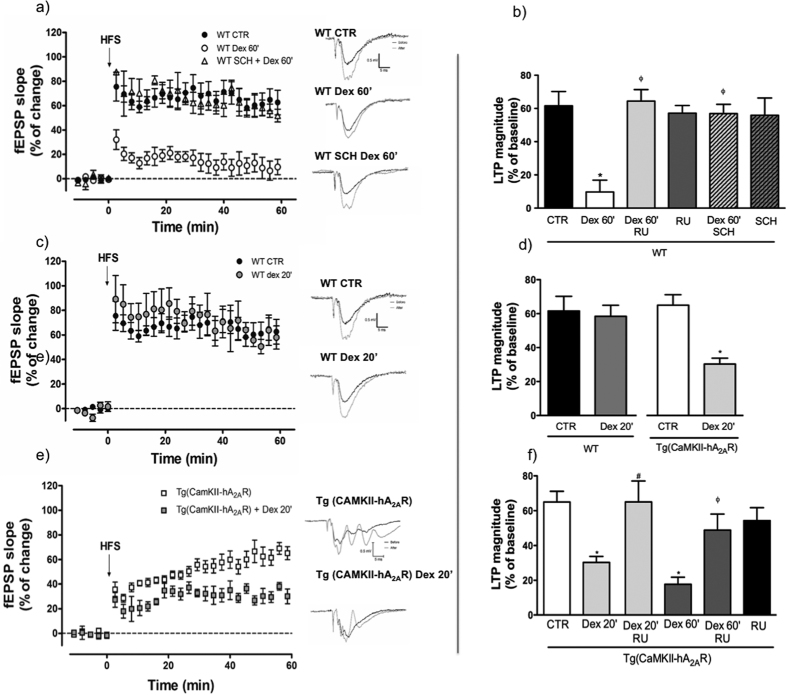
Dexamethasone induced deficits in synaptic plasticity are prevented by adenosine A_2A_ receptor (A_2A_R) blockade and exacerbated by A_2A_R overexpression. High frequency stimulation (HFS: 100 Hz, 1s) was used to evaluate synaptic plasticity in hippocampal slices. (**a**) Incubation of slices from WT rats with dexamethasone (100 nM) for 1 h decreases LTP magnitude an effect prevented by the A_2A_R antagonist SCH58261 (50 nM). (**b**) Magnitude of the effects of dexamethasone, RU486 (100 nM) and SCH58261 (n = 3–8) in hippocampal slices from WT rats. Results are presented as mean ± SEM of n experiments analyzed using a one-way ANOVA followed by a Bonferroni *post hoc test*. *P < 0.05 compared to CTR; ^ϕ^P < 0.05 compared with dexamethasone 60 min. (**c)** Incubation of slices with dexamethasone (100 nM) for 20 minutes has no effect on LTP magnitude in WT animals (n = 3–8), whereas (**e**) in Tg(CaMKII-hA_2A_R) animals is sufficient to induce a significant decrease in LTP magnitude (n = 6–9). (**d)** Bar plots of the effects of dexamethasone and (**f**) the prevention of these effects by the GR antagonist RU486 (n = 3–9). Results are presented as mean ± SEM of n experiments analyzed using an unpaired t-test for comparisons between WT and Tg(CaMKII-hA_2A_R) and one-way ANOVA followed by a Bonferroni *post hoc test* for drug effects.*P < 0.05 compared to control, ^#^P < 0.05 compared with dexamethasone 20 min, ^ϕ^P < 0.05 compared with dexamethasone 60 min.

We tested if A_2A_Rs were involved in the known GR gene-dependent effects on synaptic plasticity by applying dexamethasone for the time periods indicated, followed by one-hour resting prior to recording, to allow gene-dependent effects. Prolonged exposure (for 60 min) of WT hippocampal slices to a synthetic GR agonist, dexamethasone (100 nM), abolished long-term potentiation (LTP) (Fig. 3a,b) an effect prevented by the GR antagonist, RU486 (Fig. 3b). Interestingly, the blockade of A_2A_R with SCH58261 (50 nM) prevented these GR-induced effects as efficiently as RU486 (Fig. 3a,b), whereas neither GR nor A_2A_R antagonists alone had an effect on control (vehicle-treated) slices (Fig. 3b). We then tested whether, conversely, A_2A_R overexpression increased susceptibility to dexamethasone. Using a shorter exposure - 20 min - to dexamethasone (100 nM), we found that dexamethasone treatment had no impact on LTP magnitude in WT animals (Fig. 3c,d). In contrast, such shorter exposure time was sufficient to decrease LTP magnitude in tg(CaMKII-hA_2A_R) animals (Fig. 3d,e), an effect completely prevented by the GR antagonist RU486 (Fig. 3f).

**Figure 4:**
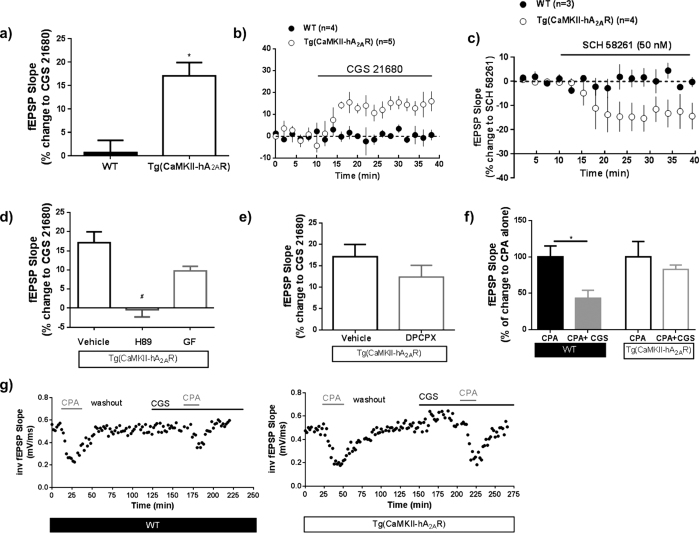
Overexpression of adenosine A_2A_ receptor (A_2A_R) induces age-like modifications in adenosine neuromodulation. (**a,b)** The A_2A_R selective agonist CGS21680, 30 nM, has an effect on basal fEPSP slope in Tg(CaMKII-hA_2A_R) but not in WT animals ; Results were analyzed using a unpaired Student’s t-test (*P < 0.05 comparing to WT); (**c**) A_2A_R tonically increase excitatory transmission in Tg(CaMKII-hA_2A_R) animals, an effect revealed by the inhibitory effect of the A_2A_R selective antagonist SCH58261 (50 nM) on basal synaptic transmission, that was not observed in WT animals. (**d**) The effect of CGS21680, 30 nM is blocked by H89 (1 μM), a PKA antagonist, but not GF (1 μM) a PKC antagonist. Results were analyzed using One-way ANOVA followed by a Bonferroni’s multiple comparison *post hoc* test (^#^P < 0.05 comparing with Tg(CaMKII-hA_2A_R with CGS21680 alone). (**e)** The effect of A_2A_R activation does not change in the presence of the selective adenosine A_1_R antagonist DPCPX (100 nM;n = 3); P > 0.05 using a paired Student’s t-test analysis). (**f**) The effect of CGS58261 upon CPA, 30 nM, on fEPSP is lost in Tg(CaMKII-hA_2A_R) animals. Results were analyzed using a paired Student’s t-test comparing to CPA alone (n = 4/7, *P < 0.01 in WT animals comparing to CPA alone). (**g**) Time course of a representative experiment testing the A_1_/A_2A_ crosstalk in WT versus Tg(CaMKII-hA_2A_R).

We further explored whether the A_2A_R signaling in these animals followed an aging-like pattern. In Tg(CaMKII-hA_2A_R) animals, A_2A_R tonically increase excitatory transmission, an effect revealed by the inhibitory effect of the A_2A_R selective antagonist SCH58261 (50 nM) on basal synaptic transmission (Fig. 4c), that was not observed in WT animals. The effect of CGS 21680 (30 nM), a selective A_2A_R agonist on basal synaptic transmission was much higher in tg(CaMKII-hA_2A_R) than in WT animals (Fig. 4a,b). This effect was abolished by H89, a protein kinase A (PKA) inhibitor, but not by GF 109203x, a protein kinase C (PKC) inhibitor (Fig. 4d). To evaluate if the tonic adenosine inhibitory tonus was altered in tg(CaMKII-hA_2A_R) animals, we activated A_2A_R while blocking A_1_R with a selective antagonist, DPCPX (50 nM). A_1_R blockade did not prevent A_2A_R effects on basal synaptic transmission (Fig. 4e). Finally we explored if the A_2A_R- A_1_R crosstalk, shown to disappear in normal aging^30^, is lost in the rats that overexpress A_2A_R. While in WT animals, A_1_R activation by CPA (30 nM) causes a strong inhibition of synaptic transmission that is attenuated when A_2A_R are simultaneously activated with CGS 21680; in tg(CaMKII-hA_2A_R) animals, the A_2A_R activation did not modify A_1_R mediated effects (Fig. 4f,g).

### Adenosine A_2A_R regulates GR transcriptional activity and translocation

**Figure 5:**
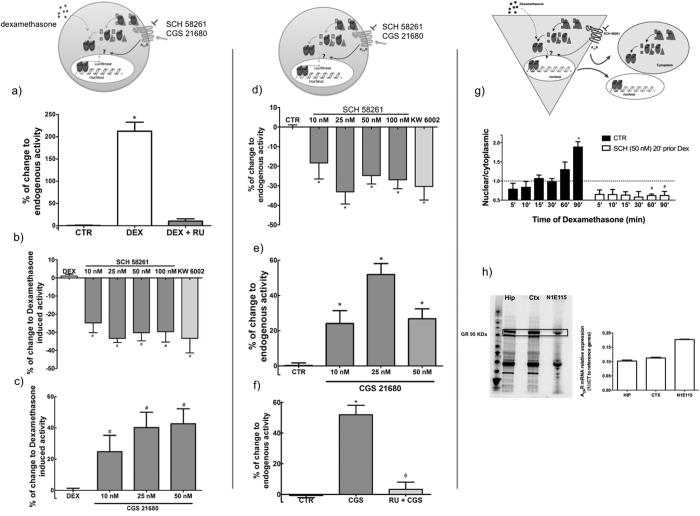
Adenosine A_2A_ receptors (A_2A_R) modulate glucocorticoid response element (GRE) regulated luciferase expression in N1E115 cells and promote dexamethasone induced Glucocorticoid Receptor (GR) translocation to the nucleus. (**a**) Dexamethasone induced an increase in luciferase activity (n =  8–17) (**b**) which is decreased upon A_2A_R blockade by two antagonists, SCH 58261 (10–100 nM) and KW 6002 (50 nM) (n = 5–11) and (**c**) increased upon direct A_2A_R activation with CGS 21680 (10–50 nM) (n = 3–9), as depicted in the upper schemes. Activation of A_2A_R alone is sufficient to modulate endogenous GR transcriptional activity (**d**) A_2A_R antagonist decreases luciferase activity (n = 6–14) while (**e**) A_2A_R agonist increases it (n = 3–11). (**f**) A_2A_R effects are prevented by the glucocorticoid receptor (GR) antagonist, RU 486 (100 nM, n = 5–10). Results are presented as mean ± SEM of n experiments. In (**d**–**f**) results were normalized to CTR, i.e., to the condition without dexamethasone, while in b) and c) results are normalized to DEX-induced activity. *P < 0.05 compared to control, ^Φ^P < 0.05 compared with dexamethasone induced luciferase activity calculated using a one-way ANOVA followed by a Bonferroni *post hoc test*. (**g**) Left panel illustrates the gradual enrichment of GR in the nuclear fraction of neuronal cultures over time of exposure to dexamethasone (n = 2–4). This increase is completely prevented by blocking A_2A_R with SCH 58261- 50 nM (in right panel). Results are presented as mean ± SEM of n experiments. *P < 0.05 compared to control, ^#^P < 0.05 compared with dexamethasone calculated using two-way ANOVA followed by a Bonferroni *post hoc*. (**h**) Expression levels of GR and A_2A_R in N1E115, compared to hippocampal (Hip) and cortical (Ctx) tissue.

We then evaluated if this increased sensitivity to dexamethasone in tg(CaMKII-hA_2A_R) animals is linked to an ability of A_2A_R to alter transcriptional regulation by GR. Therefore, mouse neuroblastoma N1E115 cells were transiently transfected with the plasmid pGL3(GRE)3_TK_Luc (GRE_Luc) that contains the glucocorticoid response elements (GRE, at which GR binds to regulate gene transcription) coupled to the luciferase gene. Exposure to dexamethasone (100 nM for 24 h) triggered the expected increase in luciferase expression, an effect blocked by the GR selective antagonist RU486 (100 nM; Fig. 5a). Notably, A_2A_R blockade with SCH58261 (10–100 nM) or KW6002, (30 nM) reduced the dexamethasone-induced increase of luciferase expression (Fig. 5b). Conversely, A_2A_R activation with the selective agonist CGS21680 (10–50 nM), increased the dexamethasone-induced luciferase expression (Fig. 5c). Finally, even in the absence of an exogenous GR activation, A_2A_R blockade decreased luciferase expression (Fig. 5d) while A_2A_R activation increased it (Fig. 5e); this effect of A_2A_R on the endogenous GR activity was prevented by RU486 (Fig. 5f).

In addition, we explored the ability of A_2A_R to control the nuclear translocation of GR in primary cortical neuronal cultures. As expected, dexamethasone (100 nM) induced a significant enrichment of GR in the nuclear *versus* cytoplasmic fraction in a time-dependent manner, which was maximal after 90 minutes; this effect was completely inhibited upon A_2A_R blockade (Fig. 5g), in line with data obtained in N1E115 cells.

### Overexpression of A_2A_R receptors downregulates the expression of GR target genes

**Figure 6:**
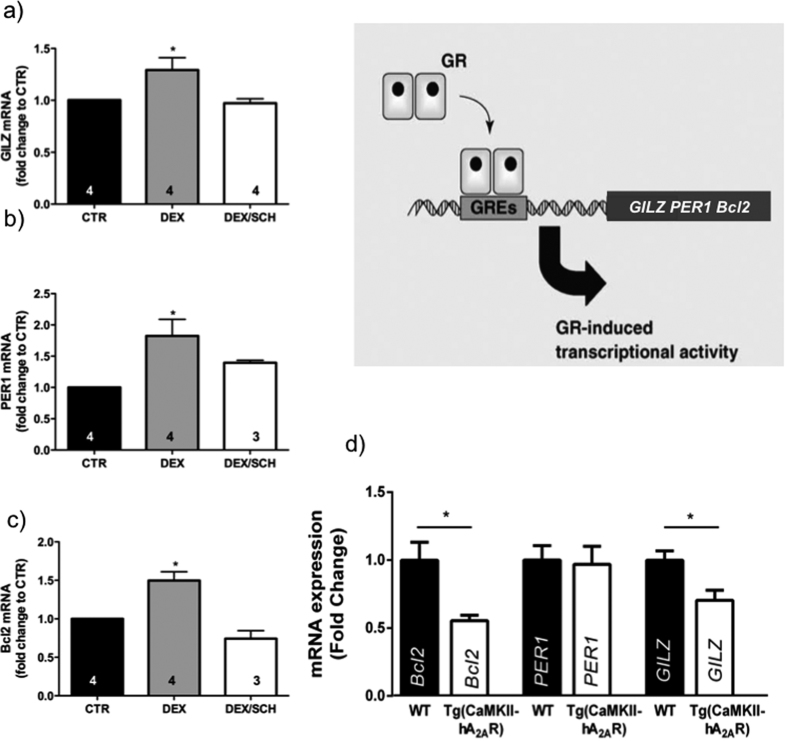
Adenosine A_2A_ receptors (A_2A_R) regulates transcription of GR target genes. mRNA levels of *GILZ* (**a**), P*ER1* (**b**) and *Bcl2* (**c**) increase in primary neurons after incubation with dexamethasone (100 nM) for 1 h an effect prevented by the presence of the selective A_2A_R antagonist SCH 51280 (50 nM), n = 3–5. *P < 0.05 compared to control, calculated using one-way ANOVA followed by a Bonferroni *post hoc* test (**d**) mRNA levels of *GILZ*, P*ER1* and *Bcl2* in the hippocampus of Tg(CaMKII-hA_2A_R) compared to WT animals. Results are presented as mean ± SEM of n experiments. *P < 0.05 compared to control, calculated using two-way ANOVA followed by a Bonferroni *post hoc*. The upper right scheme depicts the GR-mediated GRE activation to induce transcription of *GILZ*, P*ER1* and *Bcl2*.

In order to test whether A_2A_Rs are impacting on GR-dependent regulation of endogenous target genes, we evaluated the effect of selective A_2A_R inhibition on GR-dependent gene regulation in primary neuronal cultures. Quantitative PCR experiments targeting GR-activated genes (*GILZ*; *PER1* and *Bcl2*)^31^,^32^,^33^, containing GRE motifs^34^,^35^,^36^ were run following 1h incubation with dexamethasone (100 nM), in the presence or absence of the A_2A_R selective antagonist SCH51280 (50 nM). Arguing for a physiological role for A_2A_R in modulating GR activity, we found that the increased *GILZ*, *PER1* and *Bcl2* mRNA expression induced by dexamethasone was significantly reduced whenever A_2A_R are blocked with SCH 58261 (Fig. 6a–c).

Finally, we assessed the impact of the age-like GR downregulation and A_2A_R overactivation seen in the hippocampus of [tg(CaMKII-hA_2A_R)] (see Fig. 1), on the expression of the same GR-target genes (Fig. 6d). As predicted, we found that expression levels for both *GILZ* and *Bcl2* were decreased in [tg(CaMKII-hA_2A_R)] compared to WT animals, whereas for *PER1* we could not detect significant changes.

## Discussion

Our findings demonstrate for the first time that GR transcriptional activity and nuclear localization are modulated by adenosine A_2A_ receptors, thereby affecting GR function. Importantly, we here show that neuronal A_2A_R overexpression is sufficient to impair the HPA-axis function and decrease GR hippocampal levels. Furthermore, the combined evidence that A_2A_R over-expression increases the susceptibility to GR agonists on one hand, and that A_2A_R blockade prevents the detrimental synaptic effects of GR activation on the other hand suggests that A_2A_R play a critical role in the control of memory dysfunction by modulating GR expression and activation.

This is the first time that the link between the well-documented hippocampal increase in A_2A_R expression and the decrease in GR density and associated HPA-axis dysfuntion - features of aging and Alzheimer’s disease (AD) - is established. This also sustains the novel hypothesis that A_2A_R up-regulation through modulation of GR function is sufficient to trigger synaptic dysfunction and subsequent memory impairments. This novel A_2A_R-GR interaction has far-reaching implications in multiple pathologies in which corticosteroids play a pivotal role and that are alleviated by A_2A_R antagonists, notably caffeine.

Stress hormones and HPA-axis dysfunction have long been recognized as a critical feature underlying brain aging and pathology^37^. Indeed, altered cortisol levels are observed in post-traumatic stress syndrome or major depression^38^ and elevated salivary levels of cortisol were found to be correlated with poor cognitive function in a large study of humans aged 50–70 years^4^. Increased glucocorticoid activity has a predominant impact on the hippocampus, which plays an inhibitory role in regulating the HPA axis^2^ and controls mood and memory^39^. Thus, chronic exposure to glucocorticoids leads to cell death and hippocampal atrophy^40^,^41^ and is associated with memory impairment in the elderly^3^. Accordingly, recent evidence supports a pivotal role of stress hormones in neurodegenerative diseases, namely in AD^42^. This is re-enforced by the following observations: 1) administration of the GR antagonist, RU486, reverts multiple features of AD pathology^15^,^16^; 2) repeated stress worsens AD-induced deficits^43^; 3) elevated cortisol levels are associated with a faster disease progression in AD^5^; 4) systemic administration of glucocorticoids or stress potentiate memory impairments, hippocampal damage, β-amyloid formation and Tau pathology in transgenic AD mice^6^,^8^,^44^.

Interestingly, in aging or in other brain pathologies where a dysfunction of the HPA-axis is present, there is also an upsurge of A_2A_R in the hippocampus^1^,^11^ and their blockade has proven to be beneficial, but the mechanisms remained unknown^1^,^14^. We now provide the first demonstration that A_2A_R modulation of GR may be essential for the efficacy of A_2A_R blockade, a mechanism never hypothesized before.

The data we present clearly show that a specific increase in A_2A_R in forebrain neurons is able to impair the stress response system. A_2A_R neuronal overexpression disturbed HPA-axis and increased plasma corticosterone levels, providing a tentative connection between the adenosine neuromodulation system and the control of GR signaling.

Our observations are in agreement with previous reports that A_2A_R activation in a model of spinal cord injury mimicked the effects of GR activation in attenuating neuronal damage^10^. In particular, we showed that A_2A_Rs modulate GR transcriptional activity, an effect reversed by a GR antagonist (Fig. 5a–c). This decrease in GR-dependent transcriptional regulation could be a consequence of the observed effect of A_2A_R on the sub-cellular localization of GR (Fig. 5g). The reduced nuclear localization upon A_2A_R inhibition provides a straightforward explanation for the reduced GR-dependent regulation of genes involved in different functions such as apoptosis (Bcl2) and the circadian clock (PER1)^32^,^33^. Our results indeed show that the mRNA levels for both GILZ and Bcl-2 are decreased in the hippocampus of the transgenic rats as compared to WT animals, while PER1 levels remain unchanged (Fig. 6d).

PER1, being the only clock gene in the group, exhibits a robust amplitude of rhythmic expression, initiated in the SCN and PVN^45^. In all other brain regions, such as the hippocampus while still rhythmic, driven by CORT and GRE-dependent effects, have attenuated amplitudes^45^. Accordingly, other authors failed to see significant rhythmic expression of Per1 mRNA in whole hippocampus of rodents^46^. One can especulate that although we observe GR downregulation in Tg(CaMKII-hA2AR), the levels are not sufficient to disturb Per1 expression.

The recently described ability of caffeine to impact on the circadian clock^47^ strengthens our hypothesis since corticosterone circadian oscillation is tightly controlled by clock gene expression. Furthermore, we not only show that A_2A_R overexpression impairs circadian corticosterone, we also show that A_2A_R blockade was sufficient to prevent the deleterious impact of a synthetic GR agonist (dexamethasone) on hippocampal synaptic plasticity (Fig. 3a,b). This tight A_2A_R-GR interaction discloses a new view on how HPA-axis dysfunction emerges, and also supports the therapeutic utility of A_2A_R antagonists as an important alternative to GR antagonists in reestablishing HPA-axis dysfunction, which occurs in multiple clinical conditions^1^. In fact, the therapeutic interest of using selective A_2A_R antagonists against multiple pathologies is increasing and A_2A_R antagonists have been recently approved as co-adjuvant therapy for Parkinson’s disease^48^. Various studies also support the ability of caffeine and A_2A_R blockade to prevent memory impairment in various conditions^49^, and recent work revealed that caffeine can even have pro-cognitive effects^50^. A_2A_R antagonism was also proposed for the treatment of depression and anxiety-like disorders^51^ in agreement with the decreased incidence of depression in individuals consuming caffeine^52^. However, the lack of knowledge with regarding the mechanism of action of A_2A_R antagonists compromised their acceptance for clinical use. The present report shows that A_2A_R not only regulate HPA-axis function, but also directly modulate GR, which represent key findings for understanding the mechanisms by which A_2A_R antagonism is effective. Our data complete and strengthen our previous demonstration that A_2A_R blockade overcame stress effects by reestablishing the HPA-axis and GR levels in the hippocampus^1^. These findings are critical, not only for possible treatment strategies of the memory dysfunction associated with psychopathologies, but also in the context of aging and other circumstances in which the glucocorticoid response is impaired.

We have also observed that the A_2A_R synaptic responses in tg(CaMKII-hA2AR) are dependent on PKA activation and that *in vivo* administration of an A_2A_ blocker rescues the higher LTP recorded in these animals back to WT levels (panel f), Fig. 2). This supports the crucial contribution of the A_2A_-PKA interplay to LTP induction mechanisms, as proposed recently^53^. Furthermore, the features of adenosine neuromodulation evaluated in Tg(CaMKII-hA_2A_R) follow an aging-like profile. Namely, A_2A_R activation in Tg(CaMKII-hA_2A_R) animals has a direct effect in basal synaptic transmission, mediated by PKA and not PKC, and independent of A_1_R inhibition. This reproduces what is observed in aged animals^30^. Since A_1_ receptor levels (Fig. 1e) are not changed in transgenic animals, and we obtain the same A_2A_R effect on synaptic transmission regardless of A_1_R blockade with DPCPX (Fig. 4e), this does not seem to depend on endogenous adenosine levels. Taken together, the results now presented suggest that an increase in neuronal A_2A_R is sufficient to drive age-like changes also in adenosine modulation and consequent LTP impairment. In accordance, the LTP magnitude that we obtained from young adult Tg(CaMKII-hA_2A_R) animals (Fig. 2g) is similar to the LTP observed previously in aged animals^29^,^54^,^55^ which typically present memory deficits in a variety of tasks. The fact that this ‘aged LTP’ was normalized upon A_2A_R blockade^29^,^54^, in a similar way we have shown to occur now in Tg(CaMKII-hA2AR) animals, further reinforces this relationship.

Previous studies already hinted on a direct A_2A_R-HPA axis link, but data were contradictory. Global knockout of the A_2A_R was shown to impact on both plasma corticosterone levels and melanocyte stimulating hormone levels of pro-opiomelacortin expression^56^. This was mainly mediated by a profound effect on adenosine tonic modulation by the deletion of the gene during embryonic and postnatal development, which occurs both centrally and systemically. Exposure to higher levels of corticosterone in early-life, as occurs in these KO animals, induces an independent disruption of the inhibitory hippocampal-hypothalamic feedback control of corticosterone release resulting in chronic higher levels of this hormone in plasma, as we have demonstrated previously^1^. Due to the intrinsic limitations of that constitutive model, the authors could not prove whether the central effects would simply derive from knocking out A_2A_R KO in the adrenal glands. Our current model overcomes this confounding factor, since CAMKIIa activity which drives A_2A_R expression occurs only from postnatal day 4 onwards, mainly in glutamatergic neurons of the forebrain^57^, thus inducing an age-like physiological pattern of A_2A_R overexpression (Fig. 1d).

The transducing pathways by which A_2A_R trigger GR/GRE transcriptional activity remain to be elucidated. Given the complexicity of A_2A_R signaling^58^ and its engagement in numerous signalosome protein complexes^59^, this has been difficult to dissect. A_2A_R can recruit multiple signaling pathways, being the most common in the hippocampus the cAMP/PKA/CREB, PKC and MAPK pathways^60^. Our data indicate that A_2A_R effects are probably associated to the activation of PKA rather than PKC. The effects observed in GILZ and Bcl-2 expression levels in Tg(CaMKII-hA_2A_R) (Fig. 6d) may thus result either from the modulation of GR actions on the glucocorticoid response element (GRE), or reflect an independent action following A_2A_R-driven CREB activation and binding to cyclic AMP response elements (CREs). It is known that CREB is a positive activator of Bcl-2 expression in rat^61^. On the other hand, Per1 gene contains CRE responsive elements and its levels are elevated by activation of CREB^62^. If A_2A_R would act directly through CRE in our model, one would expect an up-regulation of both Bcl-2 and Per1 in Tg(CaMKII-hA_2A_R) animals. However, we observed a clear down-regulation of Bcl-2 and no differences in Per1 expression, which is consistent with a response to the GR downregulation and less GRE-mediated gene expression, rather than a CRE- activation for these two genes. Furthermore, there is no evidence of putative binding sites for CRE in the rat GILZ, neither in the literature nor in databases such as TRED (Transcriptional Regulatory Element Database). Alternatively, a direct protein kinase A (PKA) modulation of GR binding to GRE is also possible, as previously described^63^. PKA exerts a regulatory role in the activation of multiple nuclear hormone receptors. For example, it has been shown that PKA activates GR-dependent DNA binding in cotransfection studies. In addition, PKA can directly phosphorylate GR *in vitro*, enhances transcription by the retinoic acid receptor, and regulates dimerization of human estrogen receptor-α (revised in^64^) It has been shown that PKA associates with GR and potentiates GR-dependent transcription, namely that PKA attenuates GR cross-repression^64^. Our observation that tg(CaMKII-hA_2A_R) have an enhanced A_2A_R-PKA activation associated to an increased sensitivity to dexamethasone favours the latter hypothesis.

Finally, there is an apparent paradox that arises from the present study: the fact that both stress and A_2A_R upregulation decrease GR levels in the hippocampus, while simultaneously potentiating GR activation. The former is however reconciled by the fact that GR activation is an important pathway to decrease GR expression and activation effects^65^,^66^,^67^, particularly upon chronic exposure to glucocorticoids^68^ as in chronic stress or aging. Indeed, this combination of GR downregulation and increased levels of cortisol is observed upon aging: while GR mRNA is significantly reduced in several hippocampal subfields (i.e. stratum granulosum and temporal hippocampus proper) of aged cognitively impaired rats compared to young animals, their cortisol levels take significantly longer to return to baseline following an acute stressor, and this was significantly correlated with poorer spatial learning ability^18^. Therefore, by increasing GR nuclear location and GR-mediated transcription, A_2A_R not only increase the susceptibility to stress, but also, through the same pathway, contribute to the downregulation of GR. Whether GR are more prone to activation in these conditions due to increased nuclear localization, a higher affinity for DNA, or to faster kinetics, remains to be clarified. Additionally, a redistribution of GR receptors in the hippocampus was also observed after exposure to corticosteroids^68^ which may also account for a modified susceptibility upon higher circulating corticosterone levels.

In summary, our results provide the first evidence for a functional interaction between GR and A_2A_Rs, revealing that A_2A_R modulate GR transcriptional activity and nuclear localization directly affecting GR target genes. Moreover, we demonstrate that this interaction impacts GR-mediated effects on synaptic plasticity, and that A_2A_R blockade prevents the deleterious effects associated with GR activation/function. Our data also reveal that this interaction may be particularly important in situations where A_2A_R are over-activated, such as in chronic stress or aging, possibly due to an activation of the PKA pathway.

The beneficial effects of A_2A_R antagonists, namely caffeine, against cognitive impairments may be, at least partially, due to the now reported effects on GR. The expansion of this interaction to the immune response, cell proliferation, tumor response and other cellular functions that imply GR or corticosteroids use in therapeutics, could have an enormous clinical impact.

## Additional Information

**How to cite this article**: Batalha, V. L. *et al*. The caffeine-binding adenosine A_2A_ receptor induces age-like HPA-axis dysfunction by targeting glucocorticoid receptor function. *Sci. Rep.* **6**, 31493; doi: 10.1038/srep31493 (2016).

## Figures and Tables

**Table 1:** Primers used for genotyping and qPCR.

Primer	Target Gene	Organism	Forward Primer	Reverse Primer	Amplicon Size (bp)
CypA	PPIA peptidylprolyl isomerase A (cyclophilin A)	rat, human, mouse	TATCTGCACTGCCAAGACTGAGTG	CTTCTTGCTGGTCTTGCCATTCC	126
Rpl13A	Ribosomal protein L13A	rat, mouse	GGATCCCTCCACCCTATGACA	CTGGTACTTCCACCCGACCTC	130
Pgk1	Phosphoglycerate kinase 1	rat	ATGCAAAGACTGGCCAAGCTAC	AGCCACAGCCTCAGCATATTTC	103
hACTB	Human Actin-β	human	GGACTTCGAGCAAGAGATGG	AGCACTGTGTTGGCGTACAG	233
A2AH	Human Adenosine A2A Receptor	human, rat	AACCTGCAGAACGTCAC	GTCACCAAGCCATTGTACCG	245
A2A	Adenosine Receptor A2A	rat, mouse	ATTCCACTCCGGTACAATGG	AGTTGTTCCAGCCCAGCAT	115
A1	Adenosine A1 Receptor	rat	ACCTCCGAGTCAAGATCCCT	TTGGCTCTCCAGTCTTGCTC	160
Act-B	Actin-β	rat	AGCCATGTACGTAGCCAT	CTCTCAGCTGTGGTGGTGAA	228
CaMKII-hA_2A_	calmodulin-dependent protein kinase II promoter and human Adenosine Receptor A2A	transgene	GACTAAGTTTGTTCGCATCCC	GTGACACCACAAAGTAGTTGG	450
GILZ	Glucocorticoid induced Leucine Ziper	rat, mouse	AGCAGCCACTCAAACCAACC	AACGGAAACCACATCCCCTC	151
Bcl2	B-Cell Lynphoma	rat, mouse	CTGGTGGACAACATCGCTCTG	GGTCTGCTGACCTCACTTGTG	228
PER1	Period 1	rat, mouse	TCTCACAGTTCATCTTCTGGC	CTGTGAGTTTGTACTCTTGCTG	81
CRH	Corticotropin-releasing hormone (CRH)	rat	TGCCAAGGGAGGAGAAGAGAGCG	GGGCCCTGCAAGGCAGACAG	105
